# Tobacco consumption and quality of life among teachers: a bidirectional problem

**DOI:** 10.3389/fpubh.2024.1369208

**Published:** 2024-05-09

**Authors:** Pablo A. Lizana, Valentina Vilches-Gómez, Lisseth Barra, Lydia Lera

**Affiliations:** ^1^Laboratory of Epidemiology and Morphological Sciences, Instituto de Biología, Pontificia Universidad Católica de Valparaíso, Valparaíso, Chile; ^2^Programa de Magister en Ciencias Biológicas, Instituto de Biología, Pontificia Universidad Católica de Valparaíso, Valparaíso, Chile; ^3^Departamento de Kinesiología, Facultad de Medicina, Universidad de Chile, Santiago, Chile; ^4^Latin Division, Online Education, Keiser University, Fort Lauderdale, FL, United States

**Keywords:** tobacco use, smokers, quality of life, school teachers, mental health

## Abstract

**Objective:**

This study aimed to assess a bidirectional relationship between tobacco consumption and quality of life among Chilean teachers.

**Participants and methods:**

A total sample of 647 Chilean teachers was included in a cross-sectional study (71.8% female). Teachers completed a socio-demographic questionnaire, tobacco consumption habits, and the SF-36 questionnaire to assess quality of life. Logistic regression models were employed for statistical analysis of quality of life (physical component summary; mental component summary), and tobacco consumption habits, adjusted for socio-demographic characteristics.

**Results:**

A total of 34.2% of teachers were smokers, with the majority (68.7%) being under 45 years old. Smoking teachers demonstrated lower quality of life scores, particularly mental health and emotional problems dimensions, and mental component summary (*p* < 0.05) versus nonsmoking teachers. Teachers with tobacco consumption had a higher risk of low mental component summary (OR: 1.74; *p* < 0.001), and those with low mental component summary were more likely to be smokers (OR: 1.77; *p* < 0.002).

**Conclusion:**

These findings indicate that tobacco consumption adversely affects the quality of life of Chilean teachers, especially their mental health. Psychological support should be provided to help teachers cope with work stress and tobacco consumption.

## 1 Introduction

There is a broad body of background data to show that teaching is a profession practiced well beyond established pedagogical hours in educational centers. In this sense, teachers continue their jobs within their homes when they perform different job-related activities, such as class planning, reviewing homework, tests and projects, preparing class material, and seeking out new teaching strategies (1, 2). Therefore, work overload among teachers can lead to different afflictions or diseases linked with the physical realm such as voice disorders (3), musculo-skeletal disorders and/or obesity (1, 4, 5), as well as a deteriorated mental health (6–11), and decreased quality of life (QoL) among teachers (5, 12–15). In this context, the WHO defines quality of life as an individual’s perception of their cultural environment and their values concerning specific goals, standards, and expectations. This is coupled with mental and physical well-being, state, level of independence, interpersonal relationships, surroundings, and individual convictions (16). Furthermore, apart from work overload, it has been shown that long working hours and long commutes to educational centers cause QoL problems for teachers (2, 17) as they lack the time to perform other types of activities, such as spending time with their families (2, 13). In Chile, several studies have been carried out on the quality of life of teachers, and a significant deterioration has been observed, mainly among women and young teachers (4, 5, 12, 15).

It has also been reported that a habit used by teachers to reduce their stress levels is TC (18) since it boost energy and reduces anxiety (19). Additionally, a considerable prevalence of CT has been found in younger and female teachers (6, 20). TC is a major cause of death worldwide, with around 7 million people dying annually due to direct TC (21). TC is related with different non-transmissible diseases, including cardiovascular diseases. A large part of the deaths (70%) related with cardiovascular diseases were attributed to modifiable risk factors including TC (22). TC has also been related with different types of cancer, including in the lungs, larynx, mouth, and esophagus, as well as chronic respiratory diseases and diabetes (23). TC has been related with deficient mental and physical health as well (24–27) since TC leads to premature skin aging, tooth loss, and increased gum disease risk, along with making wounds take longer to heal (23). TC can be related with mental illnesses too, since nicotine acts on neurotransmitter pathways, affecting serotonin release, which can cause depression (21). Reports indicate that people presenting some type of mental illness have a higher probability of TC, as it has been observed that an important of the mentally ill population smokes (25, 28–31).

Across Latin America, Chile has the highest rate of TC, reaching 38.7% in 2015, compared to Argentina at 22.6% and Brazil with a 14.3% rate (32). While the National Health Survey (ENS 2016–17) indicated a TC rate of 33.3% (33), this is still a high figure and must be considered. At the gender level, men have a higher TC rate than women (43.4% men, 36.5% women) while in the age group breakdown, the age groups of 20–29 years and 30–49 years have the highest TC rate (41.1 and 41.4%) compared to other groups (33), meaning that Chile has a sustained high TC rate. In this sense, few studies have reported the prevalence of TC in Chilean teachers. Thus, a prevalence between 31.96 and 35.9% has been described in Chilean teachers (6, 20, 34).

TC has been reported as having a negative effect on QoL, as different studies have observed that smokers have a lower QoL compared with nonsmokers (35–39). Additionally, studies on teachers report a strong relationship between mental health and CT. However, there remains a reasonable doubt whether a CT affects the mental health of teachers or whether low teacher mental health increases the risk of CT. Therefore, we set out three objectives for the following research. (1) To describe the prevalence of Chilean teachers who smoke, (2) to evaluate a bidirectional association between tobacco use and mental component of QoL.

## 2 Materials and methods

### 2.1 Participants and data collection procedure

The target population of this cross-sectional study consisted of Chilean teachers working in various educational centers belonging to the three national macrozones of Chile: the north, the center, and the south (*N* = 249,865) (40). The schools were chosen randomly from 28 schools in three Chilean regions, namely: northern zone: Arica and Parinacota Region (41%); central zone: Valparaíso Region (36%); and southern zone: Araucanía Region (23%). The sample was calculated with 95% confidence and 5% error. To calculate sample size, we selected the variable with the greatest variance for this study group according to extant literature. The sample was determined with Chilean teachers’ TC and QoL variables. The minimum sample was 537 participants, where the sample size also rose by 30% in case of possible abandonment. Sampling was done between 2018 and 2019. Thus, the final sample comprised of 647 teachers (71.8% women), 409 have less than 45 years old (63.2%), 316 was married/partnered (48.8%), 407 have not children (64.2%), 407 have a contact in an indefinite-term (64.1%), 366 are teachers in private subsidized schools (56.6%).

All procedures in this study complied with bioethical standards according to the Declaration of Helsinki and were approved by the Ethics Committee of the Pontifical University of Valparaiso, Chile (n°BIOEPUCV-H 160-2017). The research was conducted between 2018–2019.

Before data collection, the establishments chosen randomly from the three macro-zones of Chile were contacted to describe the study’s objectives through a face-to-face meeting. Subsequently, each participant had to read and sign an informed consent form inviting voluntary and confidential participation in the study, which did not imply remuneration, compensation, or conflict of interest with the researchers. The inclusion criteria of this research are that the teachers are working in the classroom. Therefore, teachers who performed administrative tasks were excluded. The teachers completed the questionnaires in person and on paper. All the evaluations were carried out in the same educational establishments.

### 2.2 Instruments

The sociodemographic data of the teachers in this study were gathered via surveys, where the docents themselves provided information about their age, gender, marital status, number of children, work contract types (fixed-term or indefinite) and the type of school where they worked (public, charter school, or private school).

To evaluate teachers’ QoL, we used the SF-36 questionnaire, in the version validated for use in Chile (12, 41), since the SF-36 survey was originally created and standardized for the USA (42, 43). In addition, the SF-36 questionnaire has been validated for Chilean teachers (12), as it is widely used in them (4, 5, 15). The SF-36 questionnaire evaluates participants’ QoL via 36 Likert-type questions grouped into eight scales: physical function (PF), physical role (PR), body pain (BP), general health perception (GH), vitality (V), social function (SF), emotional role (ER), and mental health (MH). These eight dimensions are grouped into two summary measurements: The Physical Component Summary (PCS) as the first component, and the Mental Component Summary (MCS) as the second. The scores obtained from each scale and component were transformed into a scale from 0 to 100, which will be standardized calculating a T-score value for each scale and PCS and MCS measurement (43). When the T-Score values are above 50, they indicate a good QoL perception, while T-Score values below 50 indicate poor QoL perception. Considering the internal consistency of the SF-36 scale, the Cronbach’s Alpha coefficient was α ≥ 0.85 for each of the eight variables.

To evaluate teachers’ TC, we used a tobacco addiction questionnaire with simple questions classifying participants into different TC categories (6).

For our purposes, anyone who met the following criteria was considered a smoker:

- Occasional smoker: someone who smokes less than one cigarette per day.
- Daily smoker: someone who has smoked at least one cigarette per day in the last 6 months.

Teachers who responded affirmatively to the questions were classified as “smokers,” and those who responded negatively were classified as “nonsmokers.”

### 2.3 Statistical analyses

Data analysis was done with STATA 16 software for Windows. For the associations done between the categorical variables, we used Fisher’s exact test and the χ^2^ test. The participants’ age was classified into two categories (≤44 years and ≥ 45 yrs) according to the cutoff scores in the Chilean National Health Survey of 2009–2010 (44). Sociodemographic variables were evaluated between the various TC categories (non-smoker, ex-smoker, and smoker) using the χ^2^ test. We applied an ANOVA as well to evaluate the differences between the 8 QoL dimensions regarding the different TC categories, followed by a post-hoc test (Bonferroni). Two logistic regression models were done after this, the first of which was a logistic regression using the PCS and MCS from QoL (for this dichotomous variable the cut-off point was the t-score at 50 of QoL) as a dependent variable to evaluate the association with TC (smokers). The second logistic regression used tobacco-consuming teachers (smokers) as a dependent variable to evaluate whether smoking teachers tended to present lower PCS and MCS scores due to TC. The aforementioned regression models were adjusted for the gender and age covariables (gender and age variables have been selected because previous reports have identified differences in these variables in Chilean teachers) (6, 20), and the goodness of fit used for each logistic regression model was demonstrated with a Hosmer-Lemeshow test.

## 3 Results

Table 1 presents the sociodemographic characteristics analyzed by participants’ gender. A total of 647 teachers were analyzed of which 465 were women (71.8%) and 182 were men (28.1%). 63.2% of participants were in the first age category (<45 years). There is a significant association between marital status and participants’ gender (*p* < 0.005), with a higher rate among the category for men with a spouse or partner, reaching 53.8%. No significant association was observed between gender and age, having any children, work contract type, and the type of school where the participants worked.

**Table 1:** Teachers’ sociodemographic characteristics by gender.

	Total (*n* 647)		Male (*n* 182)		Female (*n* 465)		
Variables	*n*	%	*n*	%	*n*	%	*p* value^a^
Age (years)							
<45	409	63.21	115	63.19	294	63.23	0.993
>45	238	36.79	67	36.81	171	36.77	
Civil status							
Single	275	42.50	78	42.86	197	42.37	
Married/partnered	316	48.84	98	53.85	218	46.88	0.008
DWW*	56	8.66	6	3.30	50	10.75	
Children							
Have	227	35.80	70	40.00	157	34.20	0.174
Have not	407	64.20	105	60.00	302	65.80	
Type of contract							
Fixed-term	228	35.91	55	31.07	173	37.77	0.115
Indefinite-term	407	64.09	122	68.93	285	62.23	
Type of school							
Public (state)	220	34.00	54	29.67	166	35.70	0.145
Private (subsidized)	366	56.57	114	62.64	252	54.19	
Private (non-subsidized)	61	9.43	14	7.69	47	10.11	

a Chi-squared test. *p* < 0.05.

DWW, divorced/widow/widower*.

The association between sociodemographic characteristics and MSD with teachers’ QoL appears in Table 2. The PHC on QoL has a significant association with the p50 of MSD (*p* = 0.023). On the other hand, in the MHC of QoL, there are significant associations in age, contract type, and p50 category (*p* < 0.01). Significant associations allow us to observe that most teachers below the T-Score for MHC are those age 44 or less and indicating that they had 6 or more body regions with MSD. Teachers with indefinite work contracts also had better mental health (87%).

**Table 2:** Teachers’ sociodemographic traits analyzed by tobacco consumption category.

	Total (*n* 647)		No smokers (*n* 359)		Ex-smokers (*n* 67)		Smokers (*n* 221)		
Characteristics	*n*	%	*n*	%	*n*	%	*n*	%	*p* value^a^
Gender									
Male	182	28.13	100	27.86	24	35.82	58	26.24	0.307
Female	465	71.87	259	72.14	43	64.18	163	73.76	
Age (years)									
<45	409	63.21	222	61.84	35	52.24	152	68.78	0.035
>45	238	36.79	137	38.16	32	47.76	69	31.22	
Civil status									
Single	275	42.50	152	42.34	19	28.36	104	47.06	
Married/partnered	316	48.84	174	48.47	40	59.70	102	46.15	0.090
DWW*	56	8.66	33	9.19	8	11.94	15	6.79	
Children									
Have	227	35.80	127	35.67	17	26.15	83	38.97	0.168
Have not	407	64.20	229	64.33	48	73.85	130	61.03	
Type of contract									
Fixed-term	228	35.91	128	36.47	22	32.84	78	35.94	0.851
Indefinite-term	407	64.09	223	63.53	45	67.16	139	64.06	
Type of school									
Public (state)	220	34.00	118	32.87	21	31.34	81	36.65	
Private (subsidized)	366	56.57	209	58.22	39	58.21	118	53.39	0.814
Private (non-subsidized)	61	9.43	32	8.91	7	10.45	22	9.95	

DWW, Divorced/Widow/Widower*.

a Chi-squared test. *p* < 0.05.

Table 2 shows participants in different TC categories (non-smoker, ex-smoker, and smoker), analyzed against the participants’ sociodemographic traits. We observed a significant association between the participants from the first age category of <45 yrs. and the smoker-type TC category (68.7%, *p* < 0.05).

Table 3 compares the scores from each of the eight dimensions and the two summary measurements from the SF-36 QoL survey, analyzed with each participant TC category. The results showed significant differences between the role limitations dimensions due to emotional problems, mental health and on the MCS measurement (*p* < 0.05), observing that smoking teachers had a lower score on the aforementioned dimensions than non-smoking participants.

**Table 3:** Comparison of eight QoL scales and summary measurements by TC categories.

Quality of life (QoL)	No smokers (a)	Exsmokers (b)	Smokers (c)		
Dimensions	Mean-SD	Mean-SD	Mean-SD	*p* value^a^	Post hoc^b^
Physical function	51.42 ± 6.58	51.55 ± 7.11	51.39 ± 7.15	0.986	–
Physical problems*	49.94 ± 5.68	51.03 ± 5.37	49.77 ± 6.11	0.286	–
Bodily pain	45.05 ± 9.87	45.40 ± 9.13	43.92 ± 9.41	0.322	–
General health perceptions	47.79 ± 9.80	47.53 ± 8.71	46.15 ± 10.08	0.141	–
Vitality	48.84 ± 9.11	48.77 ± 8.20	47.31 ± 8.36	0.116	–
Social functioning	44.21 ± 10.52	45.24 ± 9.75	42.77 ± 10.98	0.146	–
Emotional problems**	49.11 ± 6.54	49.22 ± 5.91	47.70 ± 6.82	0.034	a > c
Mental health	47.79 ± 9.86	47.51 ± 10.14	45.51 ± 10.22	0.027	a > c
PCS	49.13 ± 6.47	49.64 ± 6.03	49.03 ± 6.51	0.788	
MCS	46.90 ± 9.65	46.88 ± 9.76	44.61 ± 10.07	0.019	a > c

a Chi-Squared. *p* < 0.05.

b ANOVA with post hoc comparison using Bonferroni test.

Differences group details (columns a,b, and c). Role limitations due to physical problems *. Role limitations due to emotional problems **. PCS, Physical Component Summary; MCS, Mental Component Summary. The data are presented in T-scores; scores above 50 indicate good QoL perception, while scores below 50 indicate poor QoL perception.

Table 4 is a logistic regression model, evaluating the association between the QoL summary measurements (PCS and MCS; low PCS and MCS are values under 50) and TC (smokers). Smoking teachers have a higher risk of low MCS scores (OR: 1.74; *p* < 0.05). Teachers who were < 45 years old presented a greater risk of significantly lower PCS scores (OR: 1.86; *p* < 0.01), while for teachers <45 yrs. age granted a protective factor, as they had a lower risk of low MCS scores (OR: 0.56; *p* < 0.01).

**Table 4:** Logistic regression model to evaluate the association between the PCS and MCS for QoL regarding TC (smokers), adjusted by gender and age.

	PCS (50th percentile)		MCS (50th percentile)	
	OR [95% CI]*	*p* value	OR [95% CI]*	*p* value
Tobacco consumption (smokers)	0.96 [0.68–1.33]	0.795	1.74 [1.23–2.47]	0.002
Gender	1.36 [0.96–1.93]	0.082	1.29 [0.90–1.83]	0.161
Age (<45 years)	1.86 [1.34–2.58]	0.000	0.56 [0.40–0.77]	0.000
Hosmer–Lemeshow Test^a^	0.299		0.822	

PCS, Physical Component Summary; MCS, Mental Component Summary. *OR [95% CI], Odds Ratios [95% confidence interval].

a A value above 0.05 indicates the goodness of fit of the models are satisfactory.

Table 5 contains a logistic regression with the TC category (smokers) as a dependent variable, and QoL adjusted by gender and age. Teachers with low MCS scores for QoL had a higher risk of being smokers (OR: 1.77; *p* < 0.01). This model also shows that the TC risk factor is independent of participants’ age and gender.

**Table 5:** Logistic regression to evaluate the association between TC (smoking) with the QoL summary measurements (PCS and MCS) adjusted for gender and age.

	Tobacco consumption (smokers)		
	OR [95% CI]	SE	*p* value
PCS^a^	0.89 [0.63–1.24]	0.32	0.481
MCS^b^	1.77 [1.25–2.52]	0.15	0.001
Gender	1.13 [0 0.78–1.63]	0.21	0.527
Age	0.76 [0.53–1.09]	0.14	0.131
Hosmer–Lemeshow test^c^	0.620		

a PCS, Physical Component Summary.

b MCS, Mental Component Summary; OR, Odds Ratios [confidence interval]; SE, Standard Error.

c A value above 0.05 indicates that the model fits the data.

## 4 Discussion

The main results show that TC prevalence among teachers considered in this study was 34.2%. This is lower than other studies, such as one from 2003 where the TC rate among Chilean teachers reached 35.9% (34). The decreasing TC rate appears not only among teachers, but also across Chile, falling from 39.8% in the 2009–2010 National Health Survey (ENS), to 32.5% in the 2016–2017 (33, 44). Across South America, Chile has one of the highest TC rates compared with other countries, such as Argentina at 22.5% and Colombia with 9.5% (19). However, in other countries, TC among teachers is notably lower than in Chile. TC among Turkish teachers stood at 20.1%22, while in Botswana it was only 3.2% (45).

The age of teachers in the sample mainly fell into the first age category, i.e., between 25 and 44 years old (63.21%) (Tables 1, 2). These data align with those reported by the Education Ministry in the 2020 teachers’ variation, which reported that 62.9% of teachers recorded in the Chilean school system fell within the <45 year age range (10). Our results also indicate a significant association between TC and teachers being <45 years old (*p* < 0.05). These results are similar to the data from the 2016–2017 ENS, where participants from the 25–44 year age group had a higher TC rate (33). Young teachers have been reported as being exposed to different problems related with teaching work, such as job instability, a situation which negatively impacts young teachers’ mental health given its concomitant financial uncertainty (11). We should add that young teachers are more likely to have negative mental health impacts as well since they have high anxiety and depression rates (6, 10) which could be related with the aforementioned problems.

It is widely documented that smokers tend to have a lower QoL than nonsmokers (36–38). With regards to our results, we can observe that smoking teachers tend to score lower on the role limitation dimensions due to emotional problems, mental health, and on the MCS measurement (*p* < 0.05). We also reported that teachers who smoke have a higher risk of lower MCS scores (OR: 1.74; *p* < 0.02) and that those with a low MCS score for QoL have a higher risk of being smokers (OR: 1.77; *p* < 0.01); we can thus indicate that TC can be related with mental health problems among Chilean teachers. These findings mesh with prior studies indicating that many people with mental health problems or diseases are smokers (25, 28, 29) and that TC also doubles of probability of suffering mental health issues (46).

The physical and mental health problems associated with TC in the smoking population are a widely documented situation, but they still cause alarm. Reports show that smokers who consume 1 to 3 cigarettes per day are 3 times likelier to die from lung or heart disease (26) and that having 1 to 4 cigarettes per day is associated with doubling smokers’ mortality risks, compared with nonsmokers (27). Yusuf et al. (22) reported that around 70% of deaths related with cardiovascular disease in middle-income countries, which includes Chile, were attributed to modifiable risk factors including TC. This shows the odds of improving smokers’ QoL if they quit smoking, as they would avoid generating TC-related cardiovascular diseases. It is noted that quitting smoking improves QoL (19, 24, 25) and that behavioral interventions involving both physical activity and quitting smoking simultaneously improve QoL better than only doing one of these interventions at once. Nduaguba et al. noted that ex-smokers who did physical activity had between 70 and 160% better odds of presenting higher QoL than ex-smokers who did no physical activity (39). In this sense, the relationship between TC and mental health is influenced by various factors ranging from nicotine addiction to social and environmental determinants. In that sense, addiction to nicotine, one of the main components of tobacco, has been reported to exacerbate or contribute to the development of mental health problems (29). TC can cause changes in the nervous system and interactions with psychiatric medications, complicating existing mental treatments and having critical effects on people’s QoL (30). In addition, it has been observed that people with mental problems present a high prevalence of TC (28). In this sense, teachers who are exposed to greater factors that may affect their mental health could suggest a bidirectional influence, in which mental health problems may increase tobacco use. Tobacco use, in turn, may exacerbate mental health conditions (31). In this context, it has been observed that in a bidirectional manner, teachers with high emotional exhaustion consume more tobacco, and conversely, teachers with high TC also present a higher risk of emotional exhaustion (6). Therefore, the evidence shows that health strategies in teachers cannot be treated as individual factors but must be addressed in a comprehensive manner.

The teaching profession has one of the highest workloads, as the work continues beyond the classroom, leading to physical and mental health problems for teachers (1, 7, 10, 12). When comparing teaching with other professions regarding engagement and work exhaustion, we observed that teachers had lower engagement and higher exhaustion than other professionals (8). It is thus important to apply methods and strategies to help teachers with their mental health (9) and TC (47). In this sense, the social environment and support systems are essential in TC and cessation. The presence of a supportive social network can facilitate smoking cessation efforts, whereas a lack of support can hinder them. In addition, social cues and reinforcement of smoking behavior through peer networks and the media can influence smoking (48, 49). Therefore, initiatives involving smoke-free (TC-free) environments within educational establishments involving teachers could be an opportunity to improve self-care and prevent TC-associated risks in teachers. In this sense, Chile has national plans involving the entire educational community to prevent TC (50). In the coming years, there should be an evaluation of the policies applied. In addition, intervention strategies aimed at teachers could be applied (47). However, interventions in teachers should be more comprehensive because the evidence in this work shows that the factors cannot be treated in isolation.

## 5 Limitations

The present study has various limitations which must be considered. The first limitation is common to all cross-sectional studies, in that it provides a momentary snapshot of the teachers involved and does not allow us to carry out a cause-effect relation. The second limitation was the study sample. While the teacher sample was representative, as it covered the three national macrozones of Chile (north, center, and south), this only provides a general nationwide vision of teachers’ TC and QoL. The third limitation is that the data obtained for the study were gathered before the COVID-19 pandemic, which has negatively impacted teachers’ QoL due to lockdowns and the fact of adjusting to a reality which has affected both their mental and physical health (10–13). Therefore, if a study similar to ours was done today after the height of the pandemic, it is likely that QoL values would be lower, and TC would be higher.

## 6 Conclusion

The objective of this study was to analyze TC among Chilean teachers and observe the effects of TC on teachers’ QoL. The present study reported that approximately one-third of the school teachers TC. In addition, we observed that TC negatively affects Chilean teachers’ QoL, as we can observe lower scores in various QoL dimensions including mental health and role limitations due to emotional problems, along with MCS among teachers who smoke. We reported a bilateral association between MCS and TC where teachers with TC had a higher risk of low MCS while teachers with low MCS also had a greater risk of smoking. Our results thus describe a negative effect of TC on QoL. Programs and public policies should be implemented to help teachers quit smoking, by showing the benefits which arise once they quit, along with reducing the risk factors which affect teachers’ mental health.

## Data availability statement

The raw data supporting the conclusions of this article will be made available by the authors, without undue reservation.

## Ethics statement

The studies involving humans were approved by Ethics Committee at Pontificia Universidad de Valparaíso, Chile (n°BIOEPUCV-H 160-2017). The studies were conducted in accordance with the local legislation and institutional requirements. The participants provided their written informed consent to participate in this study.

## Author contributions

PL: Conceptualization, Data curation, Formal analysis, Funding acquisition, Investigation, Methodology, Project administration, Resources, Supervision, Writing – original draft, Writing – review & editing. VV-G: Investigation, Writing – original draft, Writing – review & editing. LB: Investigation, Writing – original draft, Writing – review & editing. LL: Data curation, Formal analysis, Investigation, Methodology, Supervision, Writing – original draft, Writing – review & editing.
